# Lung tissue injury and hemodynamic effects of ventilations synchronized or unsynchronized to continuous chest compressions in a porcine cardiac arrest model^☆^

**DOI:** 10.1016/j.resplu.2023.100530

**Published:** 2023-12-20

**Authors:** Theresa M. Olasveengen, Christiane Skåre, Marianne Skjerven-Martinsen, Per Hoff-Olsen, Jo Kramer-Johansen, Fredrik Hoff Nordum, Morten Eriksen, Per Anderas Norseng, Lars Wik

**Affiliations:** a Department of Anesthesia and Intensive Care Medicine, Oslo University Hospital, Norway; b Institute of Clinical Medicine, University of Oslo, Norway; c Division of Forensic Medicine and Drug Abuse, Norwegian Institute of Public Health, Norway; d Norwegian National Advisory Unit for Prehospital Emergency Care (NAKOS), Division of Prehospital Services, Oslo University Hospital, Norway; e Department of Research and Development and Norwegian Centre for Prehospital Emergency Care (NAKOS), Oslo University Hospital, Norway; f Institute for Experimental Medical Research, Oslo University Hospital, Norway; g Norwegian National Advisory Unit for Prehospital Emergency Care (NAKOS), Oslo University Hospital, Oslo, Norway

**Keywords:** Cardiac arrest, Resuscitation, Ventilation, Cardiopulmonary resuscitation, Experimental study

## Abstract

**Aim:**

Compare lung injury and hemodynamic effects in synchronized ventilations (between two chest compressions) vs. unsynchronized ventilations during cardiopulmonary resuscitation (CPR) in a porcine model of cardiac arrest.

**Methods:**

Twenty pigs were randomized to either synchronized or unsynchronized group. Ventricular fibrillation was induced electrically and left for 1.5 minutes. Four minutes of basic chest compression:ventilation (30:2) CPR was followed by eight minutes of either synchronized or unsynchronized ventilations (10/min) during continuous compressions before defibrillation was attempted. Aortic, right atrial and intracerebral pressures, carotid and cerebral blood flow and cardiac output were measured. Airway monitoring included capnography and respiratory function monitor. Macro- and microscopic lung injuries were assessed post-mortem.

**Results:**

There were no significant differences between groups in any of the measured hemodynamic variables or inspiration time (0.4 vs. 1.0 s, p = 0.05). The synchronized ventilation group had lower median peak inspiratory airway pressure (57 vs. 94 cm H_2_O, p < 0.001), lower minute ventilation (3.7 vs. 9.4 l min^−1^, p < 0.001), lower pH (7.31 vs. 7.53, p < 0.001), higher pCO_2_ (5.2 vs. 2.5 kPa, p < 0.001) and lower pO_2_ (31.6 vs. 54.7 kPa, p < 0.001) compared to the unsynchronized group after 12 minutes of CPR. There was significant lung injury after CPR in both synchronized and unsynchronized groups.

**Conclusion:**

Synchronized and unsynchronized ventilations resulted in similar hemodynamics and lung injury during continuous mechanical compressions of pigs in cardiac arrest. Animals that received unsynchronized ventilations with one second inspiration time at a rate of ten ventilations per minute were hyperventilated and hyperoxygenated.

Institutional protocol number: FOTS, id 6948.

## Introduction

High quality cardiopulmonary resuscitation (CPR) is important to optimize outcome after sudden cardiac arrest. While the positive impact of high-quality chest compressions during cardiac arrest on haemodynamic and survival has been studied extensively,^1^, ^2^, ^3^, ^4^, ^5^, ^6^ the role of ventilation is less clear. There are important unanswered questions regarding how different ventilation and oxygenation strategies during CPR effects hemodynamics, pulmonary injuries, and outcomes after cardiac arrest.

Following the publication of a large 23 711 patient randomized controlled trail on ventilation during continuous vs. interrupted chest compressions, the importance of ventilation strategy has been increasingly recognized and debated.^7^ Although the authors were unable to demonstrate any significant difference in their primary outcome; survival to hospital discharge, there was significantly improved hospital-free survival and lower Modified Rankin Scale scores – indicating less disability and death, in the group where compressions were interrupted to deliver ventilations. The results of this large study prompted the International Liaison Committee on Resuscitation (ILCOR) to perform a broad systematic evidence evaluation of continuous vs. interrupted compressions during CPR.^8^ As ILCOR continues to recommend both strategies for professional providers, they acknowledge there is still a lack of evidence to guide a specific ventilation strategy during CPR.^9^, ^10^

Experimental studies in both humans and animals suggest blood flow may be enhanced by synchronizing positive pressure ventilations to the downstroke of chest compressions, maximizing the intrathoracic pressure.^11^, ^12^, ^13^, ^14^ However, in the clinical randomized trial that followed, survival was lower in patients where ventilations were synchronized to chest compression downstrokes compared to the conventional CPR group, and the authors concluded it was likely due to deleterious effects of the experimental technique.^15^ The previous observational data of unsynchronized ventilations during compressions had demonstrated extremely high airway pressures in humans − 60–110 cm H_2_O,^11^ providing a possible explanation to the disappointing clinical trial results. These high intrathoracic pressures are likely to occur when one or two compression downstrokes are delivered during the inspiration phase and could potentially exacerbate lung injury from CPR.

Our hypothesis was that short, interposed ventilations synchronized to the upstroke of the compression-decompression cycle would provide similar hemodynamics, but with less risk of lung tissue injury compared to asynchronous guidelines compliant ventilations with one second inspiration time.

## Methods

### Study design

We conducted a non-blinded experimental RCT in a porcine cardiac arrest model at the Institute for Experimental Medical Research, Oslo University Hospital. Random sequence was done using a computer software program. The pigs were randomized into two different ventilation modes: 1) unsynchronized guideline compliant ventilations with one second inspiration time at a rate of 10 min^−1^ (the ventilations were given at a fixed rate and duration without taking into account whether they collided with the chest compressions) and 2) synchronization of ventilation between two chest compressions at a rate of 10 min^−1^ (the inspiration phase was short enough to be delivered in-between the chest compressions). In both groups, the ventilations were given manually during continuous chest compressions without pauses for ventilation. Ventilations were monitored in real time with a respiratory function monitor to ensure the ventilations were delivered according to protocol.

The experiments were conducted in accordance with “Regulations on Animal Experimentation” under The Norwegian Animal Welfare Act and approved by Norwegian Animal Research Authority in compliance with EU Directive 2010/63/EU for animal experiments, with reporting in accordance with ARRIVE guidelines (checklist in Supplemental materials).^16^ The involved staff were certified with Federation of Laboratory Animal Sciences Associations category C.^17^

### Animal preparation

Twenty 30 ± 2 kg healthy domestic swine of either sex and 10–12 weeks of age were kept overnight with free access to food and water. On the study day the animals were sedated with a single intramuscular injection of ketamine (30 mg kg^−1^) and atropine 1 mg. After ear vein catheter insertion, bolus injections of propofol (3 mg kg^−1^) and fentanyl (10 mcg kg^−1^) were given and airway was secured by endotracheal intubation. Anesthesia was maintained with continuous infusions of propofol (10–15 mg kg^−1^h^−1^*)* and fentanyl (30–50 mcg kg^−1^h^−1^). During preparations, acetated Ringer’s solution was administered to achieve stable mean arterial pressure and heart rate.

During the preparatory phase animals were mechanically ventilated (Datex Capnomac Ultima™, Helsinki, Finland) with FiO_2_ 0.4, 14–20 breaths min^−1^, positive end-expiratory pressure of 5 cm H_2_O, and tidal volume (TV) adjusted to maintain end-tidal carbon dioxide (ETCO_2_) at 4.5–5.5 kPa. Thirty minutes prior to induction of cardiac arrest FiO_2_ was reduced to 0.21. TV, respiratory pattern and ETCO_2_ were measured continuously using a respiratory monitor (CO2SMO Plus! Model 8100, Novametrix Medical System Inc., Wallingford, CT, USA). Defibrillation pads were placed on the thorax, connected to a defibrillator, and used for electrocardiogram monitoring (LP12 Physio Control, Redmond, WA., USA). Urine was drained continuously through a cystostoma, and the intra-abdominal temperature was maintained at 38.5–39.5° C throughout the experiment.

### Instrumentation

Two 7F micro-tip pressure transducer catheters (Model SPC 470, Millar Instruments, Houston, TX, USA) were inserted; one through the left carotid artery and advanced just above the aortic valve for continuous arterial pressure monitoring, and another to the right atrium via the right external carotid vein. A 7.5F Swan-Ganz catheter (Edwards Lifesciences, Irvine, CA 92614, USA) was inserted into the right atrium via the right femoral vein and a fluid filled polyethylene catheter was inserted into the aorta from the right femoral artery, both for blood gas monitoring. An ultrasound flow meter probe (model 3SB880, Transonic Systems Inc., Ithaca, NY, USA) was applied to the right carotid artery. All visible branches of the right common carotid artery, except the internal carotid artery, were ligated. All invasive catheters were introduced using a cut down technique.

A craniotomy was performed approximately 10 mm anterior to the coronal suture and 15 mm to the left lateral side of the sagittal suture to place a laser Doppler flowmetry probe (Model 407, Perimed AB, Stockholm, Sweden) on the surface of the cerebral cortex. Care was taken to avoid placing the probe directly over visible vessels, and it was held in place at the cortical surface by a probe holder (Model PH 07–4, Perimed AB, Stockholm, Sweden) secured with dural sutures. A burr hole, 12–15 mm in diameter, was drilled in the cranium using an electric drill at low speed approximately 10 mm anterior to the coronal suture and 15 mm to the right lateral side of the sagittal suture. A 7F micro-tip pressure transducer catheter (Model SPC 470, Millar Instruments, Houston, TX, USA) was inserted 1–2 cm into cerebral cortex for intracerebral pressure monitoring. The probes cord was looped and sutured to the skin to minimize movement artifacts. Readings from the laser Doppler flowmetry probe were collected as arbitrary perfusion units that reflect volume flow in the part of the cerebral cortex just below the probe and expressed as a percentage of the values obtained during 30:2 BLS CPR.

### Experimental protocol

After completion of the surgical instrumentation, baseline blood gas analysis and registration of all hemodynamic variables were performed. Mechanical ventilation and propofol anesthesia were discontinued and ventricular fibrillation (VF) was induced by a trans-thoracic current (90 V AC for 3 s). Cardiac arrest was confirmed by ECG and blood pressure changes. The pigs were left for 1.5 minute in untreated VF followed by 30 seconds with continuous mechanical chest compressions rate 102 pr minute delivered by LUCAS2 (Stryker, Lund, Sweden). During CPR ventilation was provided manually with a self-inflating bag (Laerdal Silicone Resuscitator, Laerdal Medical AS, Stavanger, Norway). The vertical position of the piston was then adjusted to correct for initial changes in chest configuration and CPR was continued for four minutes in a 30:2 compression to ventilation pattern simulating basic life support (BLS). This was followed by eight minutes with continuous chest compressions with 10 ventilations per minute with either unsynchronous ventilations with one second inspiration time or synchronous ventilations with inspiration time limited to the “upstroke” of the chest compression. (Advanced Life Support, ALS). Defibrillation was attempted after 8 minutes. If the first defibrillation was unsuccessful, a second defibrillation was attempted after 30 seconds of CPR. Resuscitation efforts were stopped after two unsuccessful defibrillations. Animals which achieved return of spontaneous circulation were euthanized with a bolus of 20 ml intravenous Propofol 20 mg/ml and 20 ml 1 molar KCl.

### Measurement parameters

All ventilation data were measured and recorded by a respiratory function monitor (CO2SMO plus Respiratory Profile Patient monitor, Nova Metrix LLC, MA, USA). All hemodynamic variables were recorded continuously throughout the experiment (1000 Hz) using real time data acquisition hardware (NI SCXI-1000, NI PCI-6036E, National Instruments Company, TX, USA) supported with VI logger (National Instruments Company, TX, USA). Reported hemodynamic data are based on averages of the 4 minute BLS period, and 8 minute ALS period. Cardiac output was measured at one and three minutes of BLS, and one, three, five, and seven minutes of ALS using the thermodilution method with 10 ml cold saline bolus administered on the pulmonary artery catheter. Blood samples were drawn at baseline, during the last 30 seconds of the BLS period, and after eight minutes of ALS.

### Experimental outcomes

Our primary outcome variable was lung tissue injury, and our secondary outcomes were hemodynamic, ventilation and blood gas parameters. Coronary perfusion pressure (CPP) was calculated as the difference in aortic and right atrium pressures during the decompression phase. Cerebral perfusion pressure (CerPP) was calculated as the difference in aortic and intracerebral pressures during the compression phase.

### Macroscopic and light microscopic airway evaluation

After euthanasia the animals’ chests were opened. The pleural lining and the lungs were inspected in situ and thereafter harvested in toto with trachea and bronchi for macroscopic inspection. Two independent pathology researchers (MSM, PHO) blinded to randomization performed systematic macroscopic and microscopic examination of the lungs.

The following macroscopic elements were noted (yes/no): 1) Pleura: Blood in the pleural cavities? 2) Trachea and bronchi: Pale inner lining or discoloration? Empty lumina, or luminal blood or saliva? 3) Lung tissue: Gas content vs consolidation in each lobe, lacerations, traumatic bullae, oedema (fluid excess), areas of decreased ventilation, signs of postmortem hypostasis?

Lung parenchyma of each lobe was harvested for light microscopic evaluation. After formalin fixation and paraffin embedding the tissue elements were processed after routine histologic procedures. Haematoxylin eosin prepared slices were investigated by examining the slices at 5 x light microscopic magnification. Thereafter, 5 to 10 areas per lobe were inspected under 400 x light microscopic magnification. All slices were evaluated for any possible pathological changes due to inherent animal disease or inborn malformation (i.e. non traumatic pathological changes). Furthermore, signs of macroscopic consolidation were either confirmed or disconfirmed by light microscopic evaluation. Lung tissue hemorrhage was graded as either grade 1 (congestion), grade 2 (red blood cell extravasation), grade 3 (scattered red blood cells in alveolar spaces), or grade 4 (massive red blood cell content in alveolar spaces).

### Statistical analysis

Data were deemed to be normally distributed, and all variables are reported as mean ± standard deviation (SD). All statistical comparisons were done using a mean difference with 95% confidence intervals (CI) and independent samples t-test using SPSS v22 (SPSS Inc., Chicago, IL, USA). A p-value less than 0.05 was considered significant. A new experimental model was designed for the present study, and a formal power analysis was not performed. After 5 initial pilot experiments, we estimated that we would need 10 animals in each group resulting in a total of 25 animals being used.

## Results

There were no differences between the two randomized groups at baseline (Table 1) or during the first 4 minutes of BLS. After randomization and eight minutes of ALS with continuous chest compressions there were no significant differences in blood flows or perfusion pressures between the groups including cerebral perfusion pressure with synchronized ventilation (24 vs. 18 mmHg). (Table 2).

**Table 1:** Baseline hemodynamic, ventilation and blood gas parameters.

	**Ventilation method**		
	**Synchronized** **group** **(n = 10)**	**Unsynchronized group** **(n = 10)**	**Mean difference (95% CI)**
**Hemodynamic parameters:**			
Aortic Pressure (mmHg)	86 ± 16	93 ± 11	6 (−6, 19)
Right Atrial Pressure (mmHg)	6 ± 6	5 ± 3	0 (−3, 4)
Coronary Perfusion Pressure (mmHg)	81 ± 17	87 ± 11	6 (−7, 17)
Intracranial Pressure (mmHg)	10 ± 6	8 ± 3	0 (−4, 3)
Cerebral Perfusion Pressure (mmHg)	76 ± 16	85 ± 12	7 (−8, 20)
Carotid Blood Flow (ml/s)	158 ± 13	178 ± 41	18 (−11, 48)
Cardiac output (l/min)	3.1 ± 0.6	3.0 ± 0.4	−0.1 (−0.3, 0.6)

**Ventilation parameters:**			
Minute ventilation (l/min)	5.7 ± 0,8	5.1 ± 1,1	−0.6 (−1,5, 0,4)
Ventilation rate (per minute)	15 ± 2	14 ± 3	−1 (−3, 1)
Tidal volume (ml)	379 ± 15	367 ± 26	−12 (–33, 10)
Peak inspiratory pressure (mmH_2_O)	19 ± 2	18 ± 2	−1 (−3, 1)
Inspiration time (s)	1.4 ± 0,2	1.5 ± 0,3	0.1 (−0.7, 0.9)
Lung compliance (dyn)	28 ± 2	28 ± 4	0 (−3, 4)
EtCO_2_ (kPa)	4.6 ± 0.7	4.7 ± 0.9	0,1 (−0.7, 0.9)

**Blood gas parameters:**			
pH	7.47 ± 0.03	7.44 ± 0.03	−0.03 (−0.06, 0.00)
pO_2_ (kPa)	13.1 ± 4.2	11.5 ± 1.5	−1.7 (−4.7, 1.4)
pCO_2_ (kPa)	4.7 ± 0.4	4.9 ± 0.3	−0.2 (−0.1, 0.5)
BE	1.6 ± 0.8	0.5 ± 0.6	−0.5 (−3.2, 1.0)
SpO_2_	98%±1%	97%±1%	−1 (−1, 0)

Values given as means ± standard deviation. Differences between groups were assessed using mean difference with 95% confidence interval. EtCO_2_ = End-tidal CO_2_. BE = Base excess.

**Table 2:** Hemodynamic and ventilation parameters during 4 minutes of 30:2 CPR (BLS period) and during 8 minutes with ventilation during continuous chest compressions (ALS period).

	**BLS period** **(4 min)**	**ALS period** **(8 min)**		
	**30:2 CPR** **(n = 20)**	**Synchronized group** **(n = 10)**	**Unsynchronized group** **(n = 10)**	**Mean difference** **(95% CI)**
**Hemodynamic parameters:**				
Aortic Pressure (mmHg)	40 ± 13	39 ± 11	33 ± 5	−6 (−14, 2)
Right Atrial Pressure (mmHg)	40 ± 30	38 ± 24	30 ± 23	−8 (−30, 14)
Intracranial Pressure (mmHg)	17 ± 6	15 ± 6	15 ± 4	0 (−4, 5)
Carotid Blood Flow (ml/s)	80 ± 36	50 ± 26	41 ± 20	−9 (−30, 13)
Coronary Perfusion Pressure (mmHg)	21 ± 11	13 ± 5	15 ± 6	−2 (−4, 7)
Cerebral Perfusion Pressure (mmHg)	26 ± 12	24 ± 9	18 ± 4	−7 (−13, 0)
Cerebral Blood Flow (%)		94%±48	78%±39	−16% (−57, 25)
Cardiac output (l/min)	1.1 ± 0.3	1.1 ± 0.3	1.0 ± 0.4	−0.1 (−0.4, 0.2)

**Ventilation parameters:**				
Minute ventilation (l/min)	2.6 ± 0.8	3.7 ± 0.3	9.4 ± 3.9	5.7 (2.9, 8.5)
Ventilation rate (per minute)	6 ± 0	10 ± 0	10 ± 0	0
Tidal volume (ml)	461 ± 82	371 ± 34	942 ± 396	571 (291, 851)
Peak inspiratory pressure (mmH_2_O)	45 ± 7	57 ± 11	94 ± 10	37 (27, 48)
Inspiration time (s)	0.6 ± 0.4	0.4 ± 0.1	1.0 ± 0.8	0.6 (0, 1.2)
Lung compliance (dyn)	26 ± 10	34 ± 8	29 ± 11	−5 (−14, 4)
EtCO_2_ (kPa)	4.2 ± 1.1	3.7 ± 1.0	1.8 ± 0.7	−1.9 (−2.7, −1.0)

**Blood gas parameters:**				
Arterial pH	7.32 ± 0.07	7.31 ± 0.09	7.53 ± 0.08	0.22 (0.14, 0.29)
Arterial pO_2_ (kPa)	29.8 ± 20.2	31.6 ± 15.7	54.7 ± 17.7	23 (7.3, 38.9)
Arterial pCO_2_ (kPa)	6.4 ± 1.3	5.2 ± 1.5	2.5 ± 0.7	−3 (−3.8, −1.6)
Arterial BE	−2.6 ± 1.8	−7.1 ± 1.8	−6.0 ± 2.7	1 (−1, 3)
Arterial SpO_2_	97%±6	96%±9	99%±1	3 (−3, 9)
Mixed venous SvO_2_	27%±12	36%±27	35%±27	−1 (−24, 27)

Data are reported as averages of the 4 minute BLS period and 8 minute the ALS period. Values given as means ± standard deviation. Cerebral blood flow given as % of cerebral blood flow during BLS period (only measured as arbitrary unit). Differences between groups were assessed using mean difference with 95% confidence interval. EtCO_2_ = End-tidal CO_2_. BE = Base excess. Mean difference with 95% CI (confidence interval) between Synchronized and Unsynchronized groups.

The synchronized ventilation group had shorter inspiration time (0.4 vs. 1.0 s), lower median peak inspiratory airway pressure (57 vs. 94 cm H_2_O), lower minute ventilation (3.7 vs. 9.4 l min^−1^), lower pH (7.31 vs. 7.53), higher pCO_2_ (5.2 vs. 2.5 kPa) and lower pO_2_ (31.6 vs. 54.7 kPa) compared to the unsynchronized group after 12 minutes of CPR.

The synchronized ventilation group had less gross lung tissue injury visible in the thoracic cavity (0/10 vs. 4/10 of animals, p = 0.025), but slightly more severe traumatic bullae (4 vs. 3 cm, p = 0.035) compared to the unsynchronized group. There were no differences in the number of animals with gross lacerations, hemorrhage or traumatic bullae between the two groups. There were no significant differences in microscopic lung tissue injury between the two groups. (Table 3) Return of spontaneous circulation was achieved in 5/10 vs. 3/10 animals in the synchronized vs. unsynchronized groups, respectively. Additional data can be found in Supplemental materials.

**Table 3:** Gross and microscopic lung tissue damage at autopsy.

	**Ventilation method**		
	**Synchronized group** **(n = 10)**	**Unsynchronized group** **(n = 10)**	**P-value**
**Gross lung tissue damage**			
Thoracic cavity injury (y/n) Clear (no foam or blood) Foam Blood	0/10 10 0 0	4/6 6 2 2	0.025
Tracheal lacerations (y/n)	5/5	6/4	0.7
Bronchial lacerations (y/n) Grade 0: pale mucosa without content Grade 1: pale mucosa with some foam Grade 2: pale mucosa with bloody foam Grade 3: inflammation of the mucosa	7/3 3 3 3 1	6/4 4 3 2 1	0.6
Gross oedema of lung tissue (y/n)	0/10	0/10	N/A
Lacerations in lung tissue (y/n) Cumulative size of lacerations (cm)	2/8 0 (0–3)	0/10 0	0.1 0.5
Any traumatic bullae (y/n) Cumulative size of bullae (cm)	7/3 3 (0–4)	8/2 4 (1–9)	0.6 0.035
Any areas with low perfusion (y/n) Cumulative size of area low perfusion (cm)	10/0 29 (0–40)	9/1 21 (5–41)	0.3 0.2
Any lung tissue hemorrhage (y/n) Cumulative size of hemorrhage (cm)	9/1 7 (0–41)	7/3 5 (0–20)	0.3 0.6
**Microscopic lung tissue damage**			
Any microhaemorrhage (y/n) Grade 0: no microhaemorrhage Grade 1: congestion Grade 2: red blood cell extravasation Grade 3: scattered red blood cells in alveolar spaces Grade 4: massive red blood cells in alveolar spaces	9/1 1 0 0 2 7	9/1 1 0 1 4 4	1.0
Any atelectasis (y/n) Cumulative size of atelectasis (cm)	10/0 5 (0–7)	9/1 6 (3–7)	0.3 0.4

Values are given as actual numbers or median values (range). Cumulative size of lacerations, bullae, low perfusion, haemorrhage and atelectasis were calculated by adding the measured sizes for all 7 lung lobes for each animal. Comparisons were done by Peasons chi-squared for categorical data and Mann-Whitney U test for categorical data.

## Discussion

Ventilating intubated pigs during continuous chest compressions with short synchronized inspirations between two compressions vs. longer 1 second inspirations as indicated in the CPR Guidelines^9^, ^10^ provided similar hemodynamics. We were unable to confirm our hypothesis that unsynchronized ventilations would cause more lung injury. The unsynchronized, or guideline compliant, ventilations yielded excessive peak inspiratory pressures and tidal volumes, resulting in hyperventilation and hyperoxygenation in our experimental model.

### Interpretation of results and possible implications

The guidelines for both basic and advanced life support is to provide a 1 second inspiration until visible chest rise (or with 6 ml/kg tidal volume for advanced life support in the rare cases that this is monitored).^9^, ^10^ Pigs have a barrel shaped chest making it more difficult to assess chest rise, and while the inspiration time and ventilation rate was tightly controlled in this study, the resulting tidal volumes were excessive. Still, respiratory monitors are not the norm during resuscitation efforts in- or out-of-hospital, and assessment of chest rise is an imprecise method to determine tidal volume.^18^ It is possible that focusing on the more tangible recommendations on inspiration and ventilation rate promotes hyperventilation in clinical settings as well. A recent clinical observational trial suggest chest compressions during the inspiratory phase of ventilation may force air out of the lungs, causing so-called “reversed airflow”, further complicating ventilation during CPR.^19^

### Similarities and differences with previous experimental studies

The classic experimental studies from Johns Hopkins published in the 1980s targeted the thoracic pump mechanism where the aim was to increase forward blood flow during CPR by increasing the thoracic pressure in the compression phase and minimizing the thoracic pressure to allow venous return in the decompression phase.^11^, ^12^, ^20^ By performing compressions and ventilations at the same time they achieved a) higher intrathoracic pressures during compression + ventilation to drive forward flow, and b) lower intrathoracic pressures in the decompression phase to augment venous return as there were no ventilations between compressions. While this strategy improved hemodynamics in experimental models,^11^, ^12^, ^20^ survival was lower when they ultimately tested the strategy in a clinical randomized controlled trial.^15^ Any theoretical hemodynamic benefits seemed to be outweighed by unknown detrimental effects of the strategy. One possible detrimental effect could be increased lung injury from the excessive airway pressures generated by “colliding” the compressions and ventilations, and this theory was important when designing our experimental study. As lung injury was common in all animals in our study, our results still provide some support to lung injury as a potential confounder. Implications might be that future ventilation strategies during CPR need to carefully monitor and balance augmenting hemodynamics against the potential to cause lung injury.

In our study we chose to use manual technique using a self-inflating bag for both synchronized and unsynchronized ventilations. This represents the standard equipment available during advanced life support. A recent similar experimental study compared hemodynamics, gas exchange and lung injury assessed by computer tomography (CT) between pigs ventilated with asynchronous manual ventilations and a standard 30:2 compression-ventilation ratio strategy. The authors’ concern was that the severe hypoxia and hypercapnia often observed after prolonged CPR,^21^, ^22^ was exacerbated by their standard practice of transporting patients with ongoing mechanical chest compressions and asynchronous manual ventilations. However, despite a prolonged 30-minute CPR model, they did not observe any differences in arterial blood levels of oxygen, carbon dioxide and lactate between groups. Similarly, they did not observe any differences in post-mortem lung injury assessed by CT scan.^23^.

Other experimental studies have explored the effects standard vs. synchronized mechanical ventilations using novel ventilator modes. Kill and colleagues first compared two standard ventilator modes (intermittent positive-pressure ventilation and bilevel ventilation) to a ventilator mode capable of ventilation triggered by each chest compression, and found the novel mode yielded higher mean arterial pressure, better oxygenation and a normal mixed venous pH during CPR in a pig model.^24^ In a follow-up study they showed that this novel synchronized ventilation strategy sustained better perfusion for a prolonged time compared to standard mechanical ventilation.^25^ Although their studies did not include autopsies to assess lung injury, superior gas exchange at the end of the experiments would suggest synchronized ventilations did not cause more lung injury than their standard ventilation. The ideal ventilation strategies for manual and mechanical ventilation during CPR might be different, and there are important gaps in our knowledge for both settings.^26^.

### Limitations

Healthy young pigs are not directly comparable to human cardiac arrest patients that are most often older and have comorbidities. The barrel shaped chest may also alter both respiratory physiology and hemodynamics effects of ventilations during chest compressions compared to humans. Other important limitations in this study were a) the unblinded nature of the study design where only the researchers performing the autopsy could be blinded, and b) performing manual ventilations for both synchronous and unsynchronous ventilation groups introducing the potential for bias despite efforts to monitor respiratory physiology during the experiments. Lastly, performing multiple comparisons increases the risk of finding random statistically significant differences (type 2 error).

## Conclusions

Synchronized and unsynchronized ventilations resulted in similar hemodynamics and lung injury during continuous mechanical compressions of pigs in cardiac arrest. Animals that received unsynchronized ventilations with one second inspiration time at a rate of ten ventilations per minute were hyperventilated and hyperoxygenated.

## Financial support

The study was supported by strategic grants awarded Oslo Cardiopulmonary Resuscitation Research (OSCAR) Network by Oslo University Hospital, Department of Research and Development, Oslo University Hospital, South-Eastern Norway Regional Health Authority, Laerdal Foundation and Jahres Fund. The study sponsors were not involved in the study design, data collection, data analysis or interpretation of data.

## CRediT authorship contribution statement

**Theresa M. Olasveengen:** . **Christiane Skåre:** Conceptualization, Data curation, Formal analysis, Investigation, Methodology, Project administration, Writing – original draft. **Marianne Skjerven-Martinsen:** Data curation, Formal analysis, Investigation, Methodology, Writing – review & editing. **Per Hoff-Olsen:** Data curation, Formal analysis, Methodology, Supervision, Writing – review & editing. **Jo Kramer-Johansen:** Conceptualization, Methodology, Supervision, Writing – review & editing. **Fredrik Hoff Nordum:** . **Morten Eriksen:** Data curation, Investigation, Methodology, Resources, Writing – review & editing. **Per Anderas Norseng:** Data curation, Formal analysis, Methodology, Supervision, Writing – review & editing. **Lars Wik:** Conceptualization, Supervision, Writing – review & editing.

## Declaration of competing interest

The authors declare the following financial interests/personal relationships which may be considered as potential competing interests: T.M. Olasveengen: research grant from Laerdal Foundation, Board member of Laerdal Foundation. C. Skåre: Research grant from Zoll Foundation. M. Skjerven-Martinsen: None. J. Kramer-Johansen: None. F. Nordum: None, M. Eriksen: None, P. A. Norseng: None, P.H. Olsen: None, Lars Wik: Patents licensed to Zoll and Stryker. Medical advisor Stryker.

## References

[1] Paradis N.A., Martin G.B., Rivers E.P. (1990). Coronary perfusion pressure and the return of spontaneous circulation in human cardiopulmonary resuscitation. JAMA.

[2] Bobrow B.J., Clark L.L., Ewy G.A. (2008). Minimally interrupted cardiac resuscitation by emergency medical services for out-of-hospital cardiac arrest. Jama.

[3] Kellum M.J., Kennedy K.W., Barney R. (2008). Cardiocerebral resuscitation improves neurologically intact survival of patients with out-of-hospital cardiac arrest. Ann Emerg Med.

[4] Christenson J., Andrusiek D., Everson-Stewart S. (2009). Chest compression fraction determines survival in patients with out-of-hospital ventricular fibrillation. Circulation.

[5] Stiell I.G., Brown S.P., Nichol G. (2014). What is the optimal chest compression depth during out-of-hospital cardiac arrest resuscitation of adult patients?. Circulation.

[6] Cheskes S., Schmicker R.H., Verbeek P.R. (2014). The impact of peri-shock pause on survival from out-of-hospital shockable cardiac arrest during the Resuscitation Outcomes Consortium PRIMED trial. Resuscitation.

[7] Nichol G., Leroux B., Wang H. (2015). Trial of continuous or interrupted chest compressions during CPR. N Engl J Med.

[8] Ashoor H.M., Lillie E., Zarin W. (2017). Effectiveness of different compression-to-ventilation methods for cardiopulmonary resuscitation: a systematic review. Resuscitation.

[9] Olasveengen T.M., Mancini M.E., Perkins G.D. (2020). Adult basic life support: international consensus on cardiopulmonary resuscitation and emergency cardiovascular care science with treatment recommendations. Resuscitation.

[10] Soar J., Berg K.M., Andersen L.W. (2020). Adult advanced life support: 2020 international consensus on cardiopulmonary resuscitation and emergency cardiovascular care science with treatment recommendations. Resuscitation.

[11] Chandra N., Rudikoff M., Weisfeldt M.L. (1980). Simultaneous chest compression and ventilation at high airway pressure during cardiopulmonary resuscitation. Lancet.

[12] Chandra N., Weisfeldt M.L., Tsitlik J. (1981). Augmentation of carotid flow during cardiopulmonary resuscitation by ventilation at high airway pressure simultaneous with chest compression. Am J Cardiol.

[13] Koehler R.C., Chandra N., Guerci A.D. (1983). Augmentation of cerebral perfusion by simultaneous chest compression and lung inflation with abdominal binding after cardiac arrest in dogs. Circulation.

[14] Swenson R.D., Weaver W.D., Niskanen R.A., Martin J., Dahlberg S. (1988). Hemodynamics in humans during conventional and experimental methods of cardiopulmonary resuscitation. Circulation.

[15] Krischer J.P., Fine E.G., Weisfeldt M.L., Guerci A.D., Nagel E., Chandra N. (1989). Comparison of prehospital conventional and simultaneous compression-ventilation cardiopulmonary resuscitation. Crit Care Med.

[16] Percie du Sert N., Ahluwalia A., Alam S. (2020). Reporting animal research: explanation and elaboration for the ARRIVE guidelines 2.0. PLoS Biol.

[17] Guillen J. (2012). FELASA guidelines and recommendations. J Am Assoc Lab Anim Sci.

[18] Baskett P., Nolan J., Parr M. (1996). Tidal volumes which are perceived to be adequate for resuscitation. Resuscitation.

[19] Duchatelet C., Kalmar A.F., Monsieurs K.G., Hachimi-Idrissi S. (2018). Chest compressions during ventilation in out-of-hospital cardiac arrest cause reversed airflow. Resuscitation.

[20] Chandra N., Snyder L.D., Weisfeldt M.L. (1981). Abdominal binding during cardiopulmonary resuscitation in man. JAMA.

[21] Nelskylä A., Skrifvars M.B., Ångerman S., Nurmi J. (2022). Incidence of hyperoxia and factors associated with cerebral oxygenation during cardiopulmonary resuscitation. Resuscitation.

[22] Spindelboeck W., Gemes G., Strasser C. (2016). Arterial blood gases during and their dynamic changes after cardiopulmonary resuscitation: a prospective clinical study. Resuscitation.

[23] Kopra J., Litonius E., Pekkarinen P.T. (2023). Ventilation during continuous compressions or at 30:2 compression-to-ventilation ratio results in similar arterial oxygen and carbon dioxide levels in an experimental model of prolonged cardiac arrest. Intensive Care Med Exp.

[24] Kill C., Hahn O., Dietz F. (2014). Mechanical ventilation during cardiopulmonary resuscitation with intermittent positive-pressure ventilation, bilevel ventilation, or chest compression synchronized ventilation in a pig model. Crit Care Med.

[25] Kill C., Galbas M., Neuhaus C. (2015). Chest compression synchronized ventilation versus intermitted positive pressure ventilation during cardiopulmonary resuscitation in a pig model. PLoS One.

[26] Gazmuri R.J., Ayoub I. (2023). Ventilation during CPR: a challenge to guidelines and a call for research on lingering scientific gaps. Resuscitation.

