# Supplementary material for: Lung tissue injury and hemodynamic effects of ventilations synchronized or unsynchronized to continuous chest compressions in a porcine cardiac arrest model
Source: Resusc Plus. 2023 Dec 20;17:100530. doi: 10.1016/j.resplu.2023.100530 (PMC10753078; doi:10.1016/j.resplu.2023.100530)
Supplement: Supplementary Data 1 [file mmc1.pdf]

Supplemental material

Figure legends to supplemental figure.

BLS = Basic Life Support. ALS= Advanced Life Support. MAP = Mean arterial pressure. RAP = Right Atrial Pressure. ICP = Intracranial Pressure. CorPP = Coronary perfusion pressure. CPP = Cerebral Perfusion Pressure.

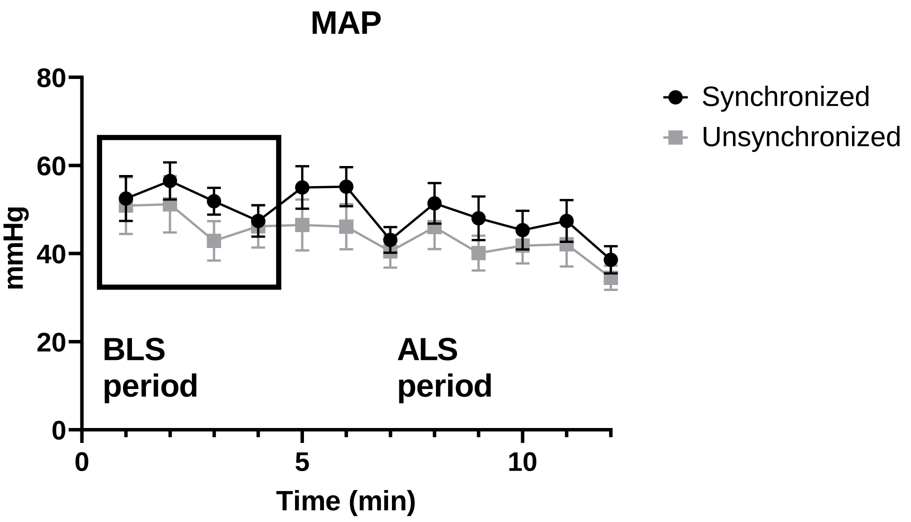
MAP
Synchronized
Unsynchronized
mmHg
BLS period
ALS period
Time (min)

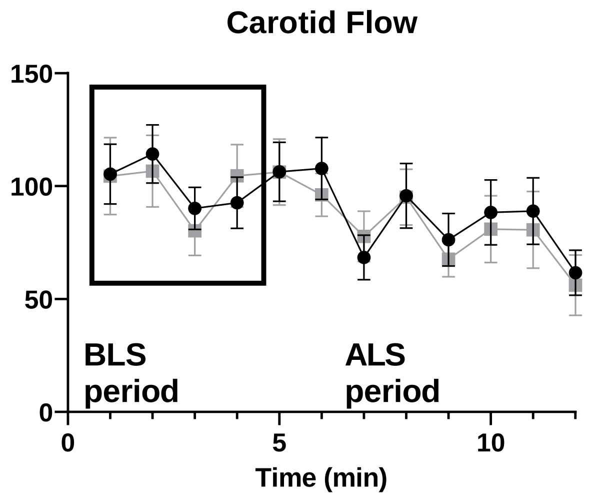
Carotid Flow
BLS period
ALS period
Time (min)

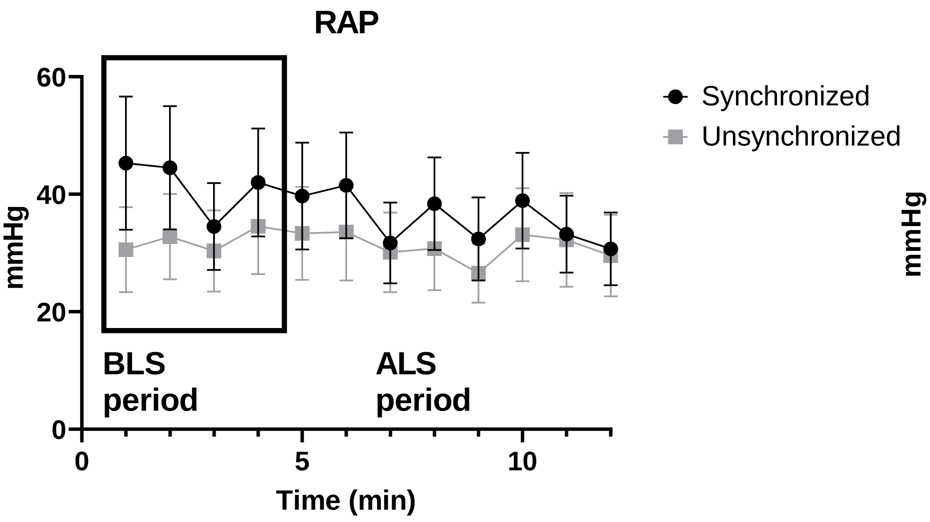
RAP
Synchronized
Unsynchronized
mmHg
BLS period
ALS period
Time (min)

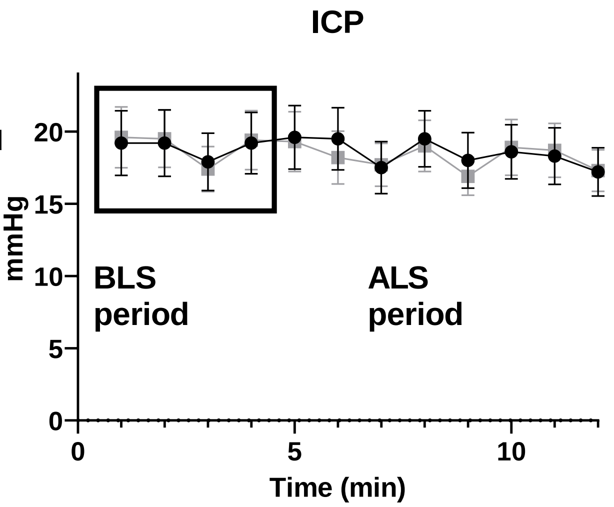
ICP
mmHg
BLS period
ALS period
Time (min)

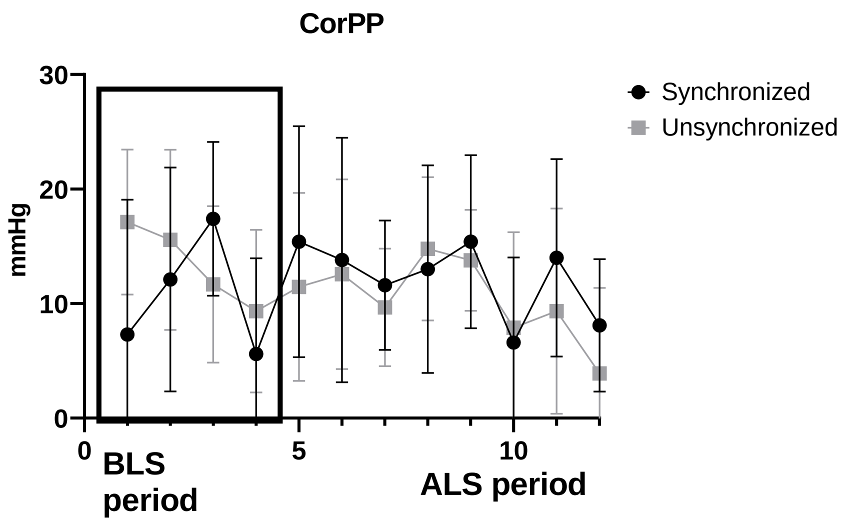
CorPP
Synchronized
Unsynchronized
mmHg
BLS period
ALS period

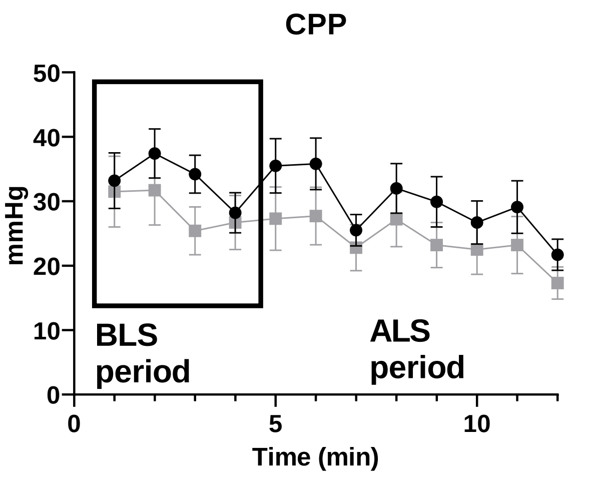
CPP
mmHg
BLS period
ALS period
Time (min)
